# Paratyphoid fever due to Salmonella enterica serotype Paratyphi A.

**DOI:** 10.3201/eid0303.970325

**Published:** 1997

**Authors:** A Kapil, S Sood, V P Reddaiah, B Das, P Seth


### Paratyphoid Fever Due to *Salmonella enterica* Serotype Paratyphi A

**To the Editor:** An outbreak of paratyphoid fever caused by *S*. Paratyphi A occurred during September and October 1996 in a residential area of New Delhi, India.

*S*. Paratyphi A has been responsible for 3% to 17% of cases of enteric fever in India (1). We suspected an outbreak because the *S*. Paratyphi A isolation rates exceeded the expected frequency based on the blood culture-positive rates from the cases of enteric fever reported by the Department of Microbiology at the All India Institute of Medical Sciences, New Delhi, the previous September and October (nine cases in 1995, 36 cases in 1996).

Thirty-six cases of culture-positive enteric fever due to *S*. Paratyphi A were reported on the basis of blood cultures received by the Department of Microbiology at the All India Institute of Medical Sciences Hospital, New Delhi, during September and October 1996. All the patients lived in the same residential area of 428 homes. The male to female ratio was 2:1, and most cases were in young adults (mean age = 20.1 yrs). All patients had a history of fever of 3 to 5 days' duration. The first culture-confirmed case was reported on September 12, 1996. After the initial case, 14 cases were reported in week 1, 10 cases in week 2, five cases in week 3, three cases in week 4, two cases in week 5, and two in week 6. Four households reported two cases each; the rest reported only one case per household. All the patients responded to ciprofloxacin treatment. All the isolates were sensitive to chloramphenicol, amoxycillin, cotrimoxazole, ciprofloxacin, gentamicin, and ceftriaxone. All the strains belonged to phage type 1.

The first suspected source of infection was contaminated food because two important Hindu festivals were celebrated on August 28 and September 5, 1996, respectively, just before the first culture-positive report on September 12. Investigators visited the affected households and distributed a questionnaire regarding demographic information, history of fever, food consumption from a common source, festival attendance, and type of water supply used. All the household contacts were also questioned. The information gathered did not indicate a foodborne outbreak. The second suspected source of infection was the water supply. The residential area receives water intermittently from a central reservoir. The water and sewage pipelines lie close to each other; the sewer line has many joints close to the water pipes, so the water may become contaminated with human excreta from the sewer line. New Delhi had a heavy rainfall toward the end of August and the beginning of September 1996, which led to waterlogging in the residential area. The contaminated soil might have entered the water pipes (because of negative pressure inside the pipes created by intermittent water supply) and contaminated the water supplied to these households. Water samples from these households during the last week of September did not contain fecal coliform. Soil samples from different sites did not contain salmonellae. Since *S. typhi* does not survive long in the environment, isolating the organism from the source is difficult by the time the outbreak is suspected (2). This may also be true for *S*. Paratyphi A.

An outbreak of enteric fever due to *S*. Paratyphi A has never been reported. Although we could not isolate the organism from the water or the soil by the time the outbreak was suspected, epidemiologic evidence suggests a waterborne outbreak.

**Arti Kapil, Seema Sood, V.P. Reddaiah, Bimal Das, and Pradeep Seth**
All India Institute of Medical Sciences,
New Delhi, India

### References

1. Saxena SN, Sen R. *Salmonella* Paratyphi A infection in India-Incidence and phage types. Trans Roy Soc Trop Med Hyg 1966;603:409-11.
2. Smith GR. Enteric infections: typhoid and paratyphoid fever. In: Wilson G, Miles A, Parker MT, editors. Principles of bacteriology, virology and immunity. 7th ed., vol. 3. London: Edward Arnold Ltd.; 1982. p. 407-33.

### MHC and Infectious Diseases

**To the Editor:** The review on the importance of the major histocompatibility complex (MHC) in infectious diseases by Singh et al. (Emerg Infect Dis 1997;3:41-9) failed to mention the potential role of human leukocyte antigen (HLA)-DM in conferring susceptibility to infectious diseases. HLA-DM is an MHC class II-like molecule essential for normal antigen processing and presentation (1). HLA-DM has been shown to function as a peptide editor, in that it influences the repertoire of peptides bound to HLA-DR.

